# Timing, frequency, and duration of incubation recesses in dabbling ducks

**DOI:** 10.1002/ece3.6078

**Published:** 2020-02-12

**Authors:** Rebecca Croston, C. Alex Hartman, Mark P. Herzog, Michael L. Casazza, Cliff L. Feldheim, Joshua T. Ackerman

**Affiliations:** ^1^ U.S. Geological Survey Western Ecological Research Center Dixon CA USA; ^2^ Suisun Marsh Program California Department of Water Resources Sacramento CA USA

**Keywords:** dabbling duck, gadwall, iButton, incubation recess, mallard, nest break, recess timing, repeatability

## Abstract

Nest attendance is an important determinant of avian reproductive success, and identifying factors that influence the frequency and duration of incubation recesses furthers our understanding of how incubating birds balance their needs with those of their offspring. We characterized the frequency and timing (start time, end time, and duration) of incubation recesses for mallard (*Anas platyrhynchos*) and gadwall (*Mareca strepera*) hens breeding in Suisun Marsh, California, USA, and examined the influences of day of year, ambient temperature at the nest, incubation day, and clutch size on recess frequency and timing using linear mixed models. Mallard, on average, took more recesses per day (1.69 ± 0.80, mean ± standard deviation) than did gadwall (1.39 ± 0.69), and 45% of mallard nest‐days were characterized by two recesses, while only 27% of gadwall nest‐days were characterized by two recesses. Mallard morning recesses started at 06:14 ± 02:46 and lasted 106.11 ± 2.01 min, whereas mallard afternoon recesses started at 16:39 ± 02:11 and lasted 155.39 ± 1.99 min. Gadwall morning recesses started at 06:30 ± 02:46 and lasted 91.28 ± 2.32 min, and gadwall afternoon recesses started at 16:31 ± 01:57 and lasted 192.69 ± 1.89 min. Mallard and gadwall started recesses earlier in the day with increasing ambient temperature, but later in the day as the season progressed. Recess duration decreased as the season progressed and as clutch size increased, and increased with ambient temperature at the nest. The impending darkness of sunset appeared to be a strong cue for ending a recess and returning to the nest, because hens returned to their nests earlier than expected when recesses were expected to end after sunset. Within hens, the timing of incubation recesses was repeatable across incubation days and was most repeatable for mallard afternoon recesses and on days in which hens took only one recess. Hens were most likely to be away from nests between 04:00 and 07:00 and between 16:00 and 19:00; therefore, investigators should search for nests between 07:00 and 16:00. Our analyses identified important factors influencing incubation recess timing in dabbling ducks and have important implications for nest monitoring programs.

## Graphical abstract

We characterized the frequency and timing (start time, end time, and duration) of incubation recesses for mallard (*Anas platyrhynchos*) and gadwall (*Mareca strepera*) hens breeding in Suisun Marsh, California, USA, and examined the influences of day of year, ambient temperature at the nest, incubation day, and clutch size on recess frequency and timing. Mallard, on average, took more recesses per day than did gadwall. Mallard and gadwall started recesses earlier in the day with increasing ambient temperature, but later in the day as the season progressed. Recess duration decreased as the season progressed and as clutch size increased, and increased with ambient temperature at the nest. The impending darkness of sunset appeared to be a strong cue for ending a recess and returning to the nest, because hens returned to their nests earlier than expected when recesses were expected to end after sunset. Our analyses identified important factors influencing incubation recess timing in dabbling ducks and have important implications for nest monitoring programs.

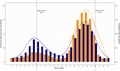

## 1 INTRODUCTION

In birds, nest attendance plays a critical role in determining nest survival (Aldrich & Raveling 1983; Prop, Eerden, & Drent, 1984). Increased nest attendance, for example, can lead to increased rates of embryonic development, which reduces the incubation period and thus minimizes both the physiological costs and the risk of depredation to both eggs and hens (Carter, Hopkins, Moore, & DuRant, 2014; Hepp, Kennamer, & Johnson, 2006; Samelius & Alisauskas, 2001). Maintaining consistent egg temperatures for the development of embryos, however, is energetically expensive (Tinbergen & Williams, 2002). The frequency, timing, and duration of absences from the nest during incubation (hereafter recesses, sensu Skutch, 1962) reflect the need of the parent to maintain the proper physical environment for egg development, while balancing their own metabolic needs (Reid, Ruxton, Monaghan, & Hilton, 2002; Tinbergen & Williams, 2002) and limiting the predation risk to both themselves and their eggs (Afton & Paulus 1992). In species in which only the female incubates, these competing needs result in patterns of nest attendance characterized by periodic incubation recesses during which the female leaves the nest unattended in order to engage in self‐maintenance activities. Incubation rhythms are typically flexible and can respond to a variety of physiological factors such as loss of fat reserves (Criscuolo, Gabrielsen, Gendner, & Maho, 2002), nest age (Brown & Fredrickson, 1987), or increases in egg cooling rates (Cooper & Voss, 2013), and environmental factors such as weather conditions (Brown & Fredrickson, 1987; Coates et al., 2016), food availability (Afton, 1980; Hohman, 1986), partial clutch loss (Ringelman & Stupaczuk, 2013), or the risk of depredation (Dassow, Eichholz, Stafford, & Weatherhead, 2012; Forbes, Clark, Weatherhead, & Armstrong, 1994).

Dabbling duck incubation is characterized by high rates of nest attendance punctuated by relatively long but infrequent (1–2 per day) recesses. Past studies have attributed variation in nest attendance and recess timing in dabbling ducks to factors including weather conditions (e.g., Afton, 1980; Caldwell & Cornwell, 1975), food availability and nutrient limitation (e.g., Afton, 1980; Ankney & Alisauskas, 1991), predator avoidance (Afton, 1980), nest site and microclimate (e.g., Caldwell & Cornwell, 1975; Ringelman, Longcore, & Owen, 1982), and nest age (e.g., Cooper & Voss, 2013; Klett & Johnson, 1982). Recesses, for example, started later in the day (Afton, 1980; Caldwell & Cornwell, 1975) and were longer (Caldwell & Cornwell, 1975; Ringelman et al., 1982) when ambient temperatures were higher, and when nest location afforded hens less efficient foraging opportunities (Ringelman et al., 1982).

Incubation behavior also is known to vary both within and among species and individuals (MacCluskie & Sedinger, 1999; Ringelman et al., 1982; Schmidt, Taylor, & Rexstad, 2005). Estimates of repeatability describe the extent to which variation within individuals contributes to the total variation in a population (Boake, 1989). High repeatability indicates that variation within individuals is substantially lower than variation among individuals, hence repeatable within an individual. Repeatability estimates are especially affected by interactions between individuals and their environment (Martin & Reale, 2008; Nussey, Wilson, & Brommer, 2007) and can provide evidence for environmental or genetic factors that explain variation in behavioral consistency (Bell, Hankison, & Laskowski, 2009).

In this study, we used nest temperature data and automated recess detection (Croston, Hartman, Herzog, Casazza, & Ackerman, 2018) to evaluate the number, timing, duration, and consistency of recesses at 942 mallard (*Anas platyrhynchos*) and gadwall (*Mareca strepera*) nests. We assessed variability in the number of recesses taken per day, and the timing (start time, end time, and duration) of incubation recesses, as a function of species, ambient temperature, clutch size, incubation day, and date. Furthermore, we examined repeatability of incubation recess timing and duration between species and morning versus afternoon recesses and modeled the probability of hen absence from the nest by hour of the day.

## 2 MATERIALS AND METHODS

### 2.1 Study site and nest monitoring

We collected dabbling duck nest temperature data during the 2015–2017 breeding seasons at the Grizzly Island Wildlife Area, Suisun Marsh, California, USA (38°8′N, 121°58′W). We located nests by searching upland fields every three weeks using nest search techniques modified from McLandress, Yarris, Perkins, Connelly, and Raveling (1996). In short, we systematically dragged a 50‐m rope, suspended between two slow‐moving all‐terrain vehicles, across the tops of vegetation, flushing any nesting hens from the disturbed vegetation. Upon discovery, we marked nests with a 2‐m bamboo stake 4 m north of the nest bowl and a vegetation‐height stake on the south edge of the nest bowl. We determined incubation stage by candling eggs (Weller, 1956). For nests found during laying, we estimated clutch completion date by counting forward, assuming one egg laid per day, until the final clutch size was reached. For nests found after the clutch was completed, we estimated clutch completion date by determining the average incubation stage of all eggs on the first visit and subtracting that value from the date the nest was found. We revisited each nest every seven days until hatching or failure. At the end of each nest visit, we re‐covered eggs with nest material, imitating female behavior at the onset of an incubation recess.

### 2.2 Measuring nest temperature

We recorded nest temperature at duck nests using iButton temperature dataloggers (Model DS1922L‐F5#, Maxim Integrated Products, Inc.). iButtons were programmed to record temperature (±0.5°C) every 4 min in 2015 and every 8 min in 2016 and 2017. For direct comparability to the 2016 and 2017 data, we censored 2015 data to 8‐min intervals.

Prior to deployment, each iButton was secured within, but protruding slightly above, the top of a large off‐white rubber stopper affixed to a long stake, which was anchored firmly into the ground. This allowed the iButton to be positioned flush with the apical surface of the eggs and facilitated direct contact with the brood patch of the incubating hen (Croston, Hartman, et al., 2018). We deployed two iButtons at each nest; one iButton was placed in the center of the nest bowl among the eggs and the second was placed immediately south of the nest bowl rim, outside of the nest, to record local ambient temperature.

### 2.3 Statistical analyses

We identified incubation recesses using an automated recess detection method as described in Croston, Hartman, et al. (2018). Briefly, we identified hen absences from the nest based on monotonic changes in nest temperature relative to each individual nest's daily variation in temperature. We adjusted the start time (−13.7 min under ambient temperature conditions >30°C or −6.9 min under ambient temperature conditions <30°C) and end time (−1.7 min) of each recess by the average time lag to detect each of these events (following Croston, Hartman, et al., 2018). From these data, we calculated the number of recesses per nest per day, the start and end times of each recess, and the duration of each recess (recess duration = recess end time—recess start time).

For analysis, we excluded data collected (a) on and prior to the clutch completion date and (b) on and after the last date that the nest was active. The last date that the nest was active was assessed through visual examination of temperature data (i.e., nest temperature tracked ambient temperature after having been relatively constant with periodic drops, indicating hen presence at the nest). For nests in which at least one egg hatched, we excluded data collected on and after the day before the estimated hatch date. Because nest visits may influence incubation behavior on the day of the visit, we also excluded data collected on days investigators visited nests, and any individual recess that began or ended on a visit day. We adjusted ambient temperature values of <3°C to a value of 3°C and >45°C to a value of 45°C, because local weather station data indicated that temperatures in excess of these values did not occur at our field site, and instead, these likely resulted from iButtons exposed to unusual conditions (e.g., the iButton may have been exposed to direct sun on an already hot afternoon). We excluded 12 nest‐days with ≥8 identified recesses (total 103 recesses), as this represented a natural break in our data, and these were likely to represent either depredation events or incomplete contact between the hen's brood patch and iButton. We also excluded 13 nest‐days with ≥5 identified recesses (total 71 recesses) when the nest became inactive on the subsequent nest‐day, as these likely represented depredation events. We did not exclude recesses which may have resulted from partial clutch depredation, as we only found partial depredations to have occurred when we subsequently visited the nest (up to one week later), and thus, we could not link partial predation events to particular recesses. Additionally, we had no way of accounting for encounters with predators that may not have resulted in the loss of eggs (Croston, Ackerman, et al., 2018). Partial depredations occurred during at least 3.8% (111 of 2,909) of all week‐long intervals in between nest visits (R. Croston, C. Alex Hartman, M. P. Herzog, M. L. Casazza, J. T. Ackerman, unpubl. data).

#### 2.3.1 Incubation recess frequency, timing, and duration

We evaluated the influence of several parameters on the number of recesses per day, recess start time, recess end time, and recess duration using linear mixed models (LMMs, R package *lme4*, Bates, Maechler, Bolker, & Walker, 2015) with restricted maximum likelihood, and with type III Wald *F* tests and Kenward–Roger degrees of freedom (R package *car*, Fox & Weisberg, 2019). For analysis of the number of recesses per day, we fit a model that included species, incubation day (days after clutch completion), mean daily ambient temperature (over 24 hrs), full clutch size (number of eggs once laying was complete), and day of year as fixed effects, with interactions of species with incubation day, mean daily ambient temperature, day of year, and full clutch size to account for possible differential effects of these parameters on mallard versus gadwall. We also included nest identification and year as random effects. For analyses of recess start time (minutes elapsed since midnight), recess end time (minutes elapsed since midnight), and recess duration (minutes), we fit separate LMMs that included species, incubation day, ambient temperature at the time the recess began (hereafter “ambient temperature”), full clutch size, and day of year (both linear and quadratic terms) as fixed effects. We included interaction terms for species with all other fixed effects. Because the distribution of recess timing was strongly bimodal, with peaks in the morning and afternoon and a natural gap occurring between the peaks around 12:00, we also included a categorical fixed effect indicating morning versus afternoon recess (“morning/afternoon,” where recess start time prior to 12:00 = “morning”) in the model predicting recess start time. For the models predicting recess end time and recess duration, we included recess start time as a circular fixed effect. We converted the recess start time (minutes elapsed since midnight) to radians by dividing by 1,440 (total minutes per day) and multiplying by 2π, and then calculated the sine (sine‐min) and cosine (cosine‐min) of these values (Zar, 1999, sensu Croston, Ackerman, et al., 2018), and included these circular time variables and their interaction with species as predictors. The model predicting recess end times excluded recesses in which the hen did not return to the nest until the subsequent calendar day. Recess duration was right‐skewed; therefore, we natural log‐transformed these data to improve normality. In all models, we included year and nest identification as random effects. We present summary results both as raw data and as model‐predicted medians and 95% prediction intervals (5th and 95th quantiles) bootstrapped over 1,000 iterations.

We observed that only approximately 2% of all recesses ended >60 min after sunset, which is a significantly lower percentage of recesses than would be expected based on the distribution of recess start times and average recess duration. This pattern suggested that hens may alter the timing of incubation recesses according to cues of impending darkness; therefore, we investigated the role that sunset may play on recess end time. We compared observed recess end times to expected recess end times that were model‐predicted using existing variation in the durations of recesses ending prior to sunset (i.e., a “not‐naïve” chance distribution of recess end times). To do this, we first fit a model identical to the one describing recess duration (see above), but which included only recesses that both began and ended prior to sunset. This allowed us to predict recess durations based on recesses that were likely unaffected by sunset. From this initial model fit, we generated predicted durations for all recesses and then calculated expected recess end times from each recess start time in our dataset (i.e., expected recess end time = observed recess start time + predicted recess duration); these became the expected recess end times to which we then compared observed recess end times.

To compare observed recess end times with expected recess end times, we fit a subsequent model in which the difference between the observed and expected recess end times (observed recess end time—expected recess end time) was treated as the response variable. Negative values indicated recess end times that were earlier than expected, and positive values indicated recess end times that were later than expected. As fixed effects, we included (a) the difference between the sunset time and the expected recess end time (sunset time—expected recess end time); negative values indicated that the expected recess end time was after sunset, (b) a categorical term indicating whether or not the recess was expected to end prior to sunset (true/false), and (c) their interaction. A significant interaction would indicate a difference in the rate at which observed recess end time changed when the expected recess end time was before versus after sunset. We included year and nest identification as random effects. We omitted recesses which began after sunset (*N* = 725, 4% of recesses), as the end times for these recesses could not have been affected by sunset time.

#### 2.3.2 Consistency and repeatability of recess timing

To examine the consistency of recess timing among days within individual hens, we first calculated the mean start time, end time, and duration of morning and afternoon recesses for each individual nest. Next, for each individual recess, we calculated the absolute difference from the mean morning or afternoon start time, end time, and duration. We also examined the consistency of hen presence versus absence from the nest by hour of the day, by calculating the proportion of each nest‐hour (hours 0–23 across all days within each nest) containing an absence from the nest (sensu Gloutney, Clark, Afton, & Huff, 1993).

We next estimated repeatability (as intraclass correlation, ICC) of recess start time, recess end time, and recess duration from linear mixed‐effects models using R package *rptR* (Stoffel, Nakagawa, & Schielzeth, 2017), which estimates repeatability using a linear mixed model framework. Repeatability describes the relative partitioning of variance in the response variable into within‐group (within‐individual) and between‐group (between‐individual) sources and can be used to quantify stable individual differences in behavior (Falconer 1981). High repeatability estimates indicate that most of the variation observed in the response variable was observed across individuals rather than within individuals. Thus, with low variation within individuals we can expect very similar behaviors for individual hens observed over time. We report the adjusted repeatability, which accounts for the variation in fixed effects by excluding the variance explained by fixed effects from the repeatability estimate, the enhanced agreement repeatability (EAR), which accounts for the variation in fixed effects by including their variation in the repeatability estimate (i.e., by including fixed effect variation in the denominator of the repeatability equation; Stoffel et al., 2017), and the proportion of variation attributable to fixed effects. We estimated standard error for each repeatability estimate using parametric bootstrapping with 1,000 replicates. We also used likelihood‐ratio tests to compare the fit of models including the grouping factor of interest (in this case, nest identification) with the fit of models constraining group‐level variance to zero (i.e., excluding the grouping factor; Stoffel et al., 2017). All repeatability estimates are based on models which include species and the morning versus afternoon categorical variable. We also included day of year (linear and quadratic) as fixed effects to account for seasonal changes in cues for recess timing, as seasonal changes in light conditions could inflate among‐individual differences in recess timing for hens breeding at different times throughout the breeding season. We estimated repeatability for each response variable using the full dataset and using subsets of the data to examine each species and recess (morning vs. afternoon) separately.

#### 2.3.3 Timing of hen absence from the nest

We modeled the likelihood of a hen being absent from the nest during each hour of the day using a binomial generalized linear mixed model (GLMM) with presence/absence as the response. We included the recess start time (circular, as sine‐hr and cosine‐hr), day of year (linear and quadratic terms), and incubation day as fixed effects. Because the distribution of recess timing showed two distinct peaks, we also included sine‐hr and cosine‐hr as 2x frequency terms (e.g., sin2x = sin(2 × radians)) to allow for the model fit to include two modes. We included interaction terms for species with day of year, incubation day, and all time variables. We included nest identification and year as random effects.

All analyses were conducted in R V 3.3.1 (R Core Team, 2019).

## 3 RESULTS

We collected 1.88 million temperature data points from 942 nests (525 mallard, 417 gadwall) between April and July 2015–2017. From these data, we identified 17,205 incubation recesses over 11,186 nest‐days (Croston et al., 2020)**.** Summary of the number of recesses per nest‐day, recess start times, recess end times, recess durations, and their variability on a population level is reported in Table 1.

**Table 1:** Summary of the number of recesses per day, and recess start times, end times, and durations for mallard and gadwall nesting in Suisun Marsh, Grizzly Island Wildlife Area, California, 2015–2017

Attribute	Species	Recess	Median	95% PI	Mean	*SD*
Number of recesses^a^	Mallard		1.70	1.65, 1.75	1.69	0.80
	Gadwall		1.41	1.36, 1.46	1.39	0.69
Recess start time^b^	Mallard	Morning	05:57	05:48, 06:06	06:14	02:46
	Gadwall	Morning	05:44	05:35, 05:53	06:30	02:46
	Mallard	Afternoon	16:42	16:33, 16:50	16:39	02:11
	Gadwall	Afternoon	16:55	16:49, 17:02	16:31	01:57
Recess end time^c^	Mallard	Morning	07:20	07:11, 07:30	08:24	03:37
	Gadwall	Morning	07:29	07:23, 07:37	08:33	03:03
	Mallard	Afternoon	19:53	19:41, 19:58	18:22	03:40
	Gadwall	Afternoon	20:13	20:08, 20:18	19:00	03:57
Recess duration^c^, ^d^	Mallard	Morning	133.08	124.80, 141.75	106.11	2.01
	Gadwall	Morning	111.75	104.61, 119.34	91.28	2.32
	Mallard	Afternoon	134.90	127.32, 143.28	155.39	1.99
	Gadwall	Afternoon	170.64	161.17, 180.21	192.69	1.89

Note

Medians and 95% prediction intervals shown are predictions generated from linear mixed models (LMMs) using parametric bootstrapping with 1,000 replicates, with all nonfocal model parameters held constant at their mean values. Means and standard deviations are generated from raw data and reflect population‐level variation in recess number and timing.

a Predictions generated for mean daily ambient temperature, day of year, and clutch size.

b Predictions generated for mean ambient temperature at the start of the recess, day of year, and clutch size.

c Predictions generated for mean ambient temperature, recess start time, day of year, and clutch size.

d Geometric mean and standard deviation shown for raw data.

### 3.1 Number of recesses per day

**Figure 1:**
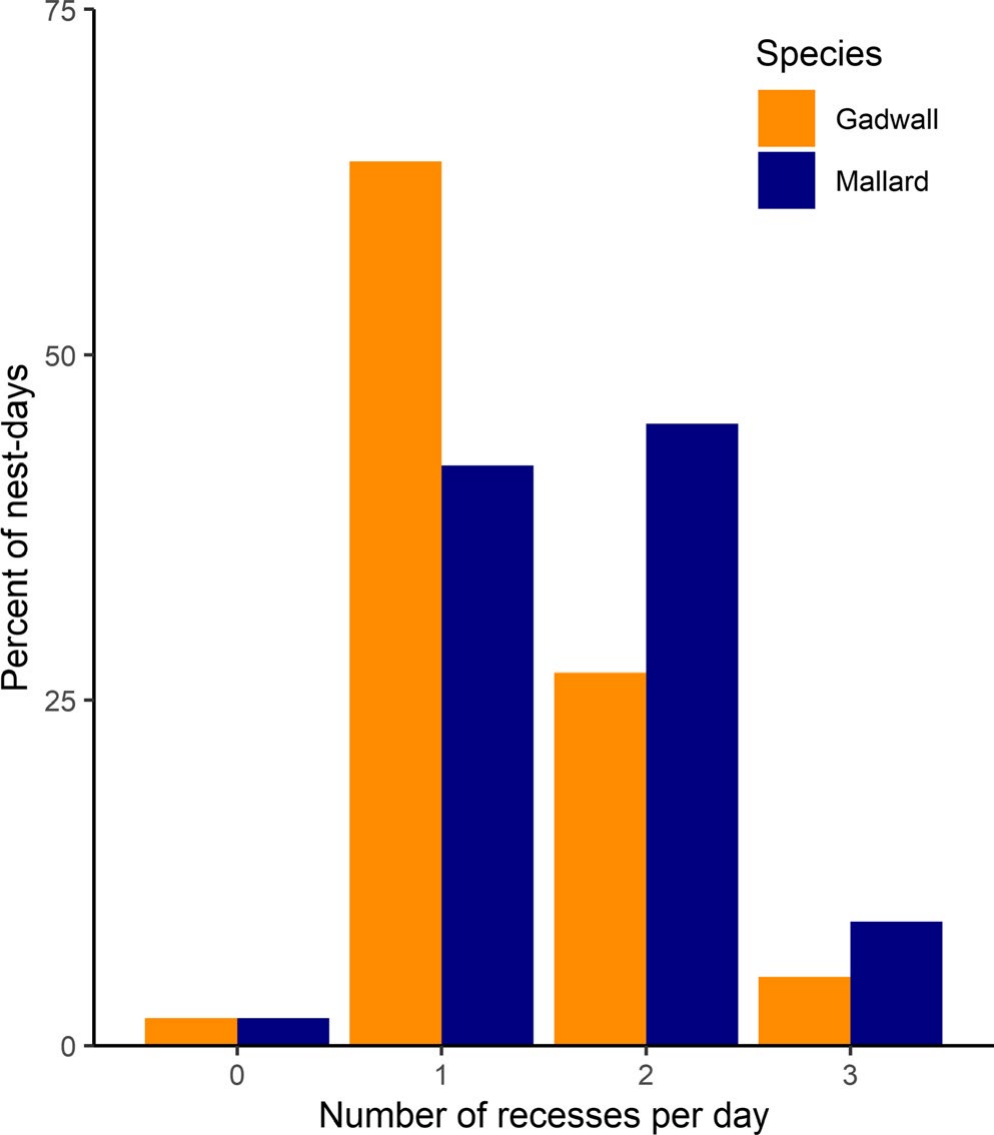
Proportion of nest‐days in which gadwall (orange bars) and mallard (blue bars) took 0, 1, 2, or 3 incubation recesses at the Grizzly Island Wildlife Area, Suisun Marsh, California, 2015–2017. Less than 2% of nest‐days included 4–7 recesses (not shown). Mallard took two recesses on a higher proportion of days than did gadwall, which more frequently took 1 recess per nest‐day

Among 11,186 nest‐days, 2% (*N* = 206) included 0 recesses, 52% (*N* = 5,797) included 1 recess, 37% (*N* = 4,152) included two recesses, 7% (*N* = 819) included three recesses, and 1% (*N* = 160) included four recesses. In total, 99.6% (*N* = 11,138) of nest‐days included ≤ 4 recesses. The remaining 0.4% of nest‐days included 5–7 recesses. Mallard took one recess on 42% of nest‐days, and two recesses on 45% of nest‐days, whereas gadwall took one recess on 64% of nest‐days and two recesses on 27% of nest‐days (Figure 1). Mallard and gadwall took recesses in the morning and afternoon, but the first recess of the day occurred in the morning during 65% (*N* = 4,066) of mallard nest‐days, and only 33% (*N* = 1,597) of gadwall nest‐days.

The number of recesses taken per day was influenced by mean daily ambient temperature at the nest and by incubation day, though the effects of these were weak even over the entire incubation period and ambient temperature range (Table 2). For gadwall, the number of recesses per day increased by 0.12 across a 26‐day incubation period (incubation day *β* = 0.0046 ± 0.0023, *F*_1,3842.46_ = 4.04, *p* = .04, Table 2). For mallard, the number of recesses per day decreased by 0.11 across a 27‐day incubation period (incubation day × species_MALLARD_ *β* = −0.0088 ± 0.0029, *F*_1,3969.35_ = 9.20, *p* < .01). Ambient temperature influenced number of recesses taken per day, and this relationship differed among species. For gadwall, the number of recesses per day increased by 0.0083 ± 0.0041 with each degree increase in mean daily ambient temperature, or 0.22 recesses per day over the entire range of mean daily ambient temperatures from 10°C to 37°C (ambient temperature *F*_1,8715.08_ = 4.10, *p* = .04). For mallard, the number of recesses per day decreased by 0.0082 ± 0.0068 with each degree increase in mean daily ambient temperature, or 0.22 recesses per day over the range of mean daily ambient temperatures (ambient temperature × species_MALLARD_ *β* = −0.017 ± 0.0055, *F*_1,10192.95_ = 9.12, *p* < .01). When all other parameters were held constant at their means, mallard took 1.70 [1.65, 1.75] (model‐predicted median [95% prediction interval]) recesses per day, whereas gadwall took 1.41 [1.36, 1.46] recesses per day (Table 1). Day of year (*F*_1,1110.74_ = 0.03, *p = *.43) and clutch size (*F*_1,1774.35_ = 0.01, *p = *.93) were not significant predictors of the number of recesses taken per day (see Table 2 for all estimates and test statistics).

**Table 2:** Summary of results from linear mixed models (LMMs) describing number of recesses per day, and recess start times, end times (including relationship between expected and observed end time relative to sunset), and durations for mallard and gadwall nesting in Suisun Marsh, Grizzly Island Wildlife Area, California, 2015–2017

Response	Fixed effects	Estimate	*SE*	Denominator *df*	F	*p*‐value
Number of recesses per day	Incubation day a	0.0046	0.0023	3,482.46	4.04	.04
	Mean daily ambient temperature	0.0083	0.0041	8,715.08	4.10	.04
	Species_MALLARD_	0.2819	0.3306	1,403.15	0.72	.39
	Day of year	−0.0003	0.0014	1,110.74	0.03	.43
	Clutch size	−0.0011	0.0130	1,774.35	0.01	.93
	Incubation day × species_MALLARD_	−0.0088	0.0029	3,969.35	9.20	<.01
	Mean daily ambient temperature × species_MALLARD_	−0.0165	0.0055	10,192.95	9.12	<.01
	Day of year × species_MALLARD_	0.0019	0.0017	1,178.94	1.22	.27
	Clutch size × species_MALLARD_	0.0233	0.0171	1,920.77	1.85	.17
Recess start time	Incubation day	−0.0372	0.3589	4,633.12	0.01	.92
	Ambient temperature	−6.1539	0.3226	12,428.63	359.92	<.0001
	Species_MALLARD_	−119.7741	25.7500	2,091.75	21.43	<.0001
	Day of year	1.0119	0.2495	1,638.02	16.33	.0001
	Day of year^2^	−0.0021	0.0058	2,636.36	0.13	.72
	Clutch size	−0.7624	1.7890	1,297.15	0.18	.67
	Morning/afternoon_MORNING_ b	−671.0407	5.3670	15,249.33	15,532.17	<.0001
	Incubation day × species_MALLARD_	−0.0750	0.4364	4,945.23	0.03	.86
	Ambient temperature × species_MALLARD_	4.0726	0.4205	14,457.62	93.40	<.0001
	Day of year × species_MALLARD_	−0.8458	0.2769	1,472.87	9.32	<.01
	Day of year^2^ × species_MALLARD_	0.0087	0.0069	2,356.03	1.60	.21
	Clutch size × species_MALLARD_	0.8772	2.3200	1,332.44	0.14	.71
	Morning/afternoon_MORNING_ × species_MALLARD_	25.9409	6.5970	16,221.21	15.43	.0001
Recess end time	Incubation day	1.7300	0.3071	4,318.50	31.65	<.0001
	Ambient temperature	−2.1200	0.3551	12,896.11	35.41	<.0001
	Species_MALLARD_	26.6393	23.8569	2,053.49	1.24	.26
	Day of year	1.1644	0.2331	1,761.02	24.86	<.0001
	Day of year^2^	−0.0088	0.0052	3,172.46	2.83	.09
	Clutch size	−0.0212	1.6791	1,427.51	0.0001	.99
	Sine‐min	−403.5180	2.9809	15,128.00	18,257.68	<.0001
	Cosine‐min	−114.3530	4.1931	14,723.04	741.59	<.0001
	Incubation day × species_MALLARD_	−0.3962	0.3825	4,663.41	1.07	.30
	Ambient temperature × species_MALLARD_	−0.9987	0.4590	13,947.91	4.72	.03
	Day of year × species_MALLARD_	−0.5668	0.2598	1,575.49	4.75	.03
	Day of year^2^ × species_MALLARD_	0.0197	0.0062	2,842.60	10.01	<.01
	Clutch size × species_MALLARD_	−1.7778	2.1791	1,438.12	0.66	.41
	Sine‐min × species_MALLARD_	10.8177	3.6579	15,503.83	8.73	<.01
	Cosine‐min × species_MALLARD_	−19.7812	5.1497	14,793.29	14.74	<.001
Observed—Expected recess end time	Sunset time − Expected recess end time	1.3136	0.0581	15,677.55	510.42	<.0001
	Sunset_BEFORE_ c	32.9812	4.1565	15,762.18	62.91	<.0001
	(Sunset time − Expected recess end time) × Sunset_BEFORE_	−1.3032	0.0583	15,691.01	499.81	<.0001
Log(Recess duration)	Incubation day	1.0016	0.0018	4,123.30	0.83	.36
	Ambient temperature	1.0313	0.0021	14,526.82	227.99	<.0001
	Species_MALLARD_	0.9542	0.1320	2,291.33	0.11	.74
	Day of year	0.9957	0.0014	1,777.92	9.99	<.01
	Day of year^2^	1.0000	0.0000	3,584.28	0.19	.67
	Clutch size	0.9835	0.0097	1,619.26	2.82	.09
	Sine‐min	0.8197	0.0139	16,109.82	136.36	<.0001
	Cosine‐min	0.8889	0.0206	15,697.59	25.83	<.0001
	Incubation day × species_MALLARD_	1.0011	0.0022	4,498.24	0.26	.61
	Ambient temperature × species_MALLARD_	1.0045	0.0026	15,092.94	2.87	.09
	Day of year × species_MALLARD_	0.9992	0.0015	1,589.58	0.28	.59
	Day of year^2^ × species_MALLARD_	1.0000	0.0000	3,224.68	0.10	.76
	Clutch size × species_MALLARD_	0.9930	0.0127	1,659.27	0.30	.58
	Sine‐min × species_MALLARD_	1.1966	0.0249	16,260.61	74.36	<.0001
	Cosine‐min × species_MALLARD_	1.2499	0.0357	15,702.55	60.82	<.0001

Note

Parameters under each response were fit in a single global model for that response. All models include nest identification and year as random effects.

a Days after clutch completion.

b Indicates whether recess began before or after 12:00.

c Indicates whether the recess was expected to end before or after sunset. Sunset_BEFORE_ indicates that sunset was prior to the expected recess end time.

### 3.2 Recess start times

**Figure 2:**
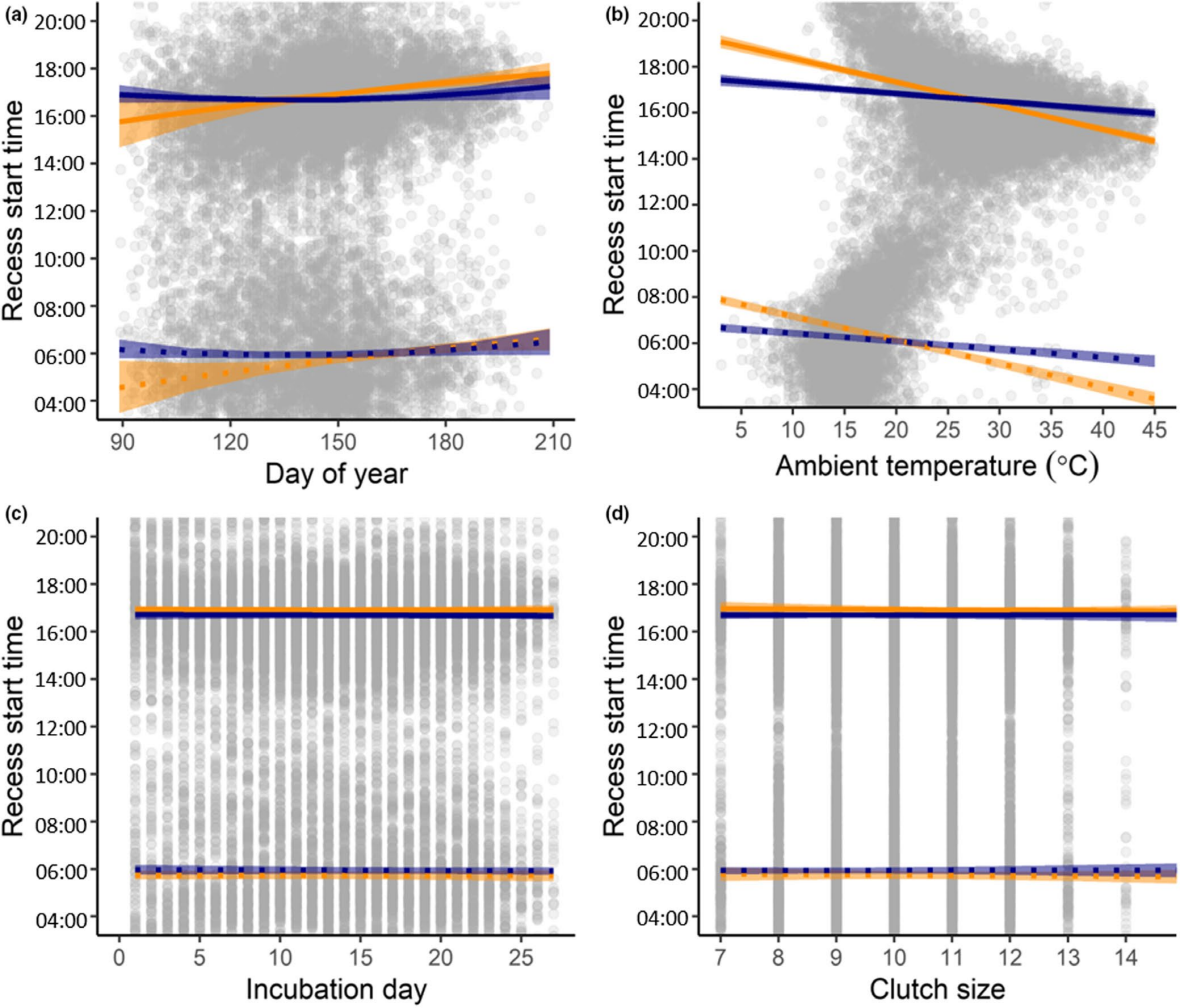
Predicted start times of morning (dotted lines) and afternoon (solid lines) incubation recesses as a function of (a) day of year (March 30 = 90 in 2016, 91 in 2015 and 2017), (b) ambient temperature at the start of the recess, (c) incubation day, and (d) clutch size for gadwall (orange lines) and mallards (blue lines) at the Grizzly Island Wildlife Area, Suisun Marsh, California, 2015–2017. Predictions are bootstrapped over 1,000 iterations and shown at the mean values of the nonfocal parameters for each panel for morning and afternoon recesses for each species, from a linear mixed model (LMM) which included incubation day, ambient temperature, species, day of year (linear and quadratic terms), clutch size, and morning/afternoon as fixed effects, with interactions between species and all other fixed effects. Year and nest identification were included as random effects. Shaded ribbons represent 95% prediction interval. Gray circles represent partial residuals calculated from LMM using R package *effects* (Fox & Weisberg, 2019) and illustrate population‐wide variation in recess start time after controlling for variation associated with all other factors in the model

Recess start times were influenced by day of year and ambient temperature, and differed among species for both morning and afternoon recesses (Table 2). When all other parameters were held constant at their mean values, the model‐predicted median start time for mallard morning recesses was 05:57 [05:48, 06:06] and for mallard afternoon recesses was 16:42 [16:33, 16:50] (Table 1). The model‐predicted median start time for gadwall morning recesses was 05:44 [05:35, 05:35] and for gadwall afternoon recesses was 16:55 [16:49, 17:02] (Table 1). For gadwall, recesses started 1.01 ± 0.25 min later each day the season progressed (day of year *F*_1_,_1638.02_ = 16.33, *p* = .0001). For mallard, recesses started 0.16 ± 0.37 min later each day the season progressed (day of year × species_MALLARD_ *β* = −0.85 ± 0.28, *F*_1,1472.87_ = 9.32, *p* < .01, Figure 2a). For gadwall, recesses started 6.15 ± 0.32 min earlier per 1°C increase in ambient temperature (ambient temperature *F*_1,12428.63_ = 359.92, *p* < .0001), and for mallard, recesses started 2.08 ± 0.53 min earlier per 1°C increase in temperature (ambient temperature × species_MALLARD_ *β* = 4.07 ± 0.42 *F*_1,14457.62_ = 93.40, *p < *.0001; Figure 2b). Recess start time was not influenced by incubation day (*F*_1,4633.12_ = 0.01, *p* = .92, Figure 2c) or clutch size (*F*_1,1297.15_ = 0.18, *p = *.67, Figure 2d).

### 3.3 Recess end times

Recess end times were influenced by incubation day, recess start time, day of year, and initial ambient temperature, and the effects of recess start time, day of year, and ambient temperature on recess end time differed between species (Table 2). When all other parameters were held constant at their means, the model‐predicted median end time for mallard morning recesses was 07:20 [07:11, 07:30] and for mallard afternoon recesses was 19:53 [19:41, 19:58] (Table 1). The model‐predicted median end time for gadwall morning recesses was 07:29 [07:23, 07:37] and for gadwall afternoon recesses was 20:13 [20:08, 20:18] (Table 1). Recesses ended 1.73 ± 0.31 min later in the day for each day that incubation progressed (incubation day *F*_1,4318.50_ = 31.65, *p* < .0001). Each 1°C increase in ambient temperature corresponded to recesses ending 2.12 ± 0.36 min earlier for gadwall (ambient temperature *F*_1,12896.11_ = 35.41, *p* < .0001, Table 2), and 3.12 ± 0.58 min earlier for mallard (ambient temperature × species_MALLARD_ *β* = −1.00 ± 0.46, *F*_1,13947.91_ = 4.72, *p* = .03, Table 2). For gadwall, recesses ended 130 min later at the end than at the beginning of the breeding season (day of year *β* = 1.16 ± 0.23, *F*_1,1761.02_ = 24.86, *p* < .0001), whereas for mallard, recesses ended 69 min later at the end than at the beginning of the breeding season (day of year × species_MALLARD_ *β* = −0.57 ± 0.26, *F*_1,1575.49_ = 4.75, *p* = .03; day of year^2^ × species_MALLARD_ *β* = 0.020 ± 0.0062, *F*_1,2842.60_ = 10.01, *p* < .01). Recesses that started before 05:00 or after 17:00 ended relatively earlier with later start times (negative slope), and gadwall both started and ended such recesses later than mallard (sine‐min × species_MALLARD_ *β* = 10.82 ± 3.66, *F*_1,15503.83_ = 8.73, *p* < .01; cosine‐min × species_MALLARD_ *β* = −19.78 ± 5.15, *F*_1,14793.29_ = 14.74, *p* < .01). Conversely, recesses that started between 05:00 and 17:00 ended relatively later with later start times (positive slope; sine‐min *β* = −403.52 ± 2.98, *F*_1,15128.00_ = 18,257.68, *p* < .0001; cosine‐min *β *= −114.35 ± 4.19, *F*_1,14723.04_ = 4.72, *p* < .0001). Clutch size (*F*_1,1427.51_ = 0.0001, *p* = .99) did not influence recess end time.

#### 3.3.1 Effect of sunset on recess end time

**Figure 3:**
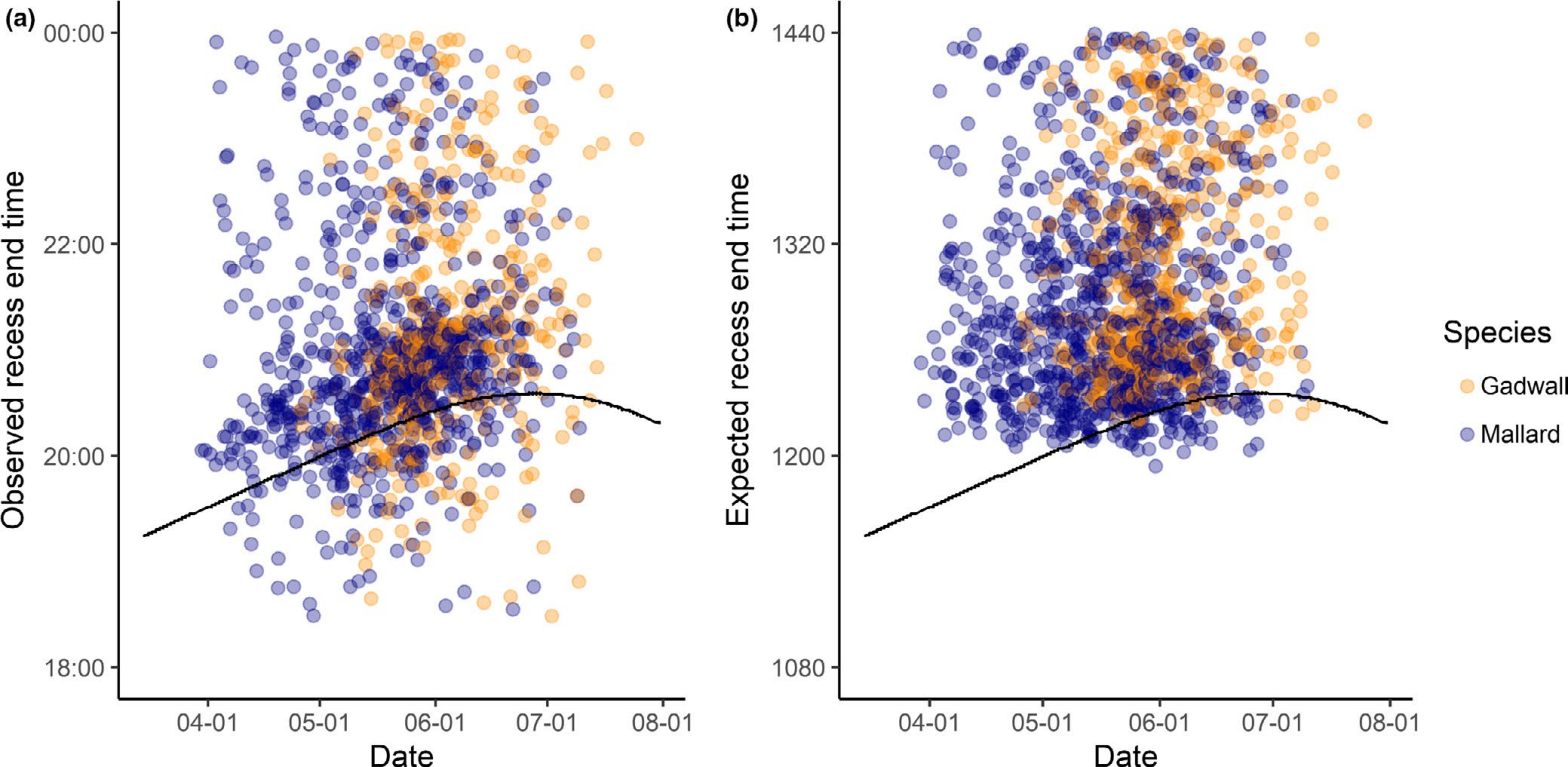
(a) Observed versus (b) expected end times for gadwall (orange circles) and mallard (blue circles) recesses at the Grizzly Island Wildlife Area, Suisun Marsh, California, 2015–2017. Data shown are restricted to recesses occurring late enough in the day to be affected by sunset (black line) time (beginning within 1 mean + S.D. duration prior to sunset time for that calendar day). Overnight recesses are not shown. Observed recess end times (a) clustered around sunset, whereas (b) expected recess end times generated by adding predicted recess durations from a linear mixed model (LMM) to observed recess end times, did not

Based on the average duration of recesses, and considering only recesses that occurred late enough in the day to potentially be affected by sunset, we expected approximately 12% of recesses to end within 30 min of sunset, but we found that approximately 22% of recesses ended within 30 min of sunset (Figure 3). Recesses that were expected to end after sunset (the difference between sunset and expected recess end time is negative) ended earlier than expected ([sunset time—expected recess end time] × sunset_BEFORE_ *β* = −1.30 ± 0.06, *F*_1,15691.01_ = 499.81, *p* < .0001, Table 2). In other words, when sunset came before a hen's expected return time, she returned to her nest earlier than expected. Our model predicted that a hen that was expected to return 30 min after sunset would return 54 min earlier than expected. A hen that was expected to return 60 min after sunset would return 94 min earlier than expected.

### 3.4 Recess durations

**Figure 4:**
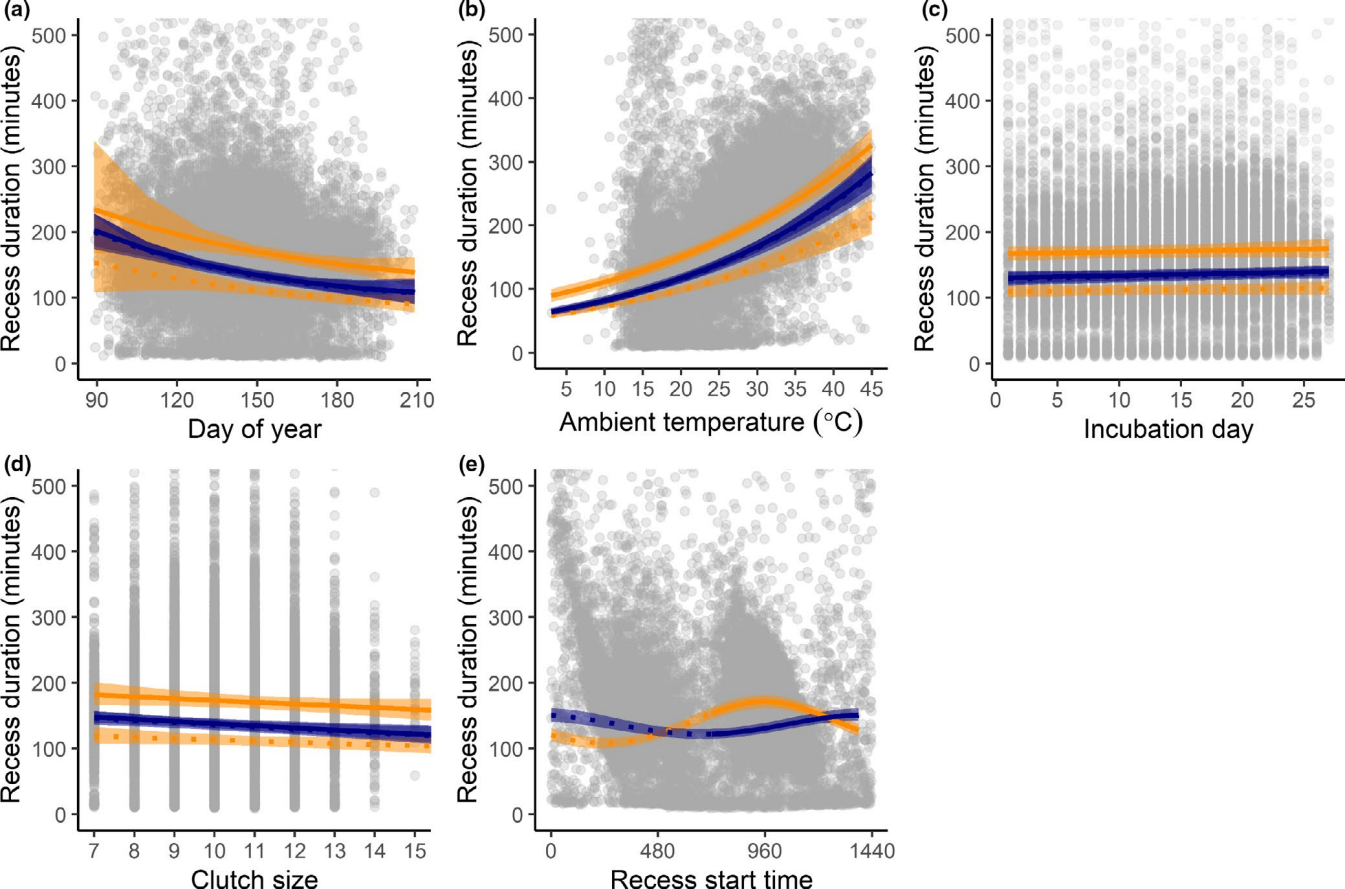
Predicted durations of morning (dotted lines) and afternoon (solid lines) recesses as a function of (a) day of year (March 30 = 90 in 2016, 91 in 2015 and 2017), (b) ambient temperature at the start of the recess, (c) incubation day, (d) clutch size, and (e) recess start time for gadwall (orange lines) and mallard (blue lines) at the Grizzly Island Wildlife Area, Suisun Marsh, California, 2015–2017. Predictions are bootstrapped over 1,000 iterations and shown at the mean values of the nonfocal parameters for each panel, for morning and afternoon recesses for each species from a linear mixed model (LMM) which included species, incubation day, ambient temperature, day of year (linear and quadratic terms), and recess start time (circular; sine‐min and cosine‐min) as fixed effects, with interactions between species and all other fixed effects. Year and nest identification were included as random effects. Shaded ribbons represent 95% prediction interval. Gray circles represent partial residuals calculated from LMM using R package *effects* (Fox & Weisberg, 2019) and illustrate population‐wide variation in recess duration after controlling for variation associated with all other factors in the model

Recess duration was influenced by the day of year, ambient temperature, and recess start time, and the relationship between start time and duration differed among species. When all other parameters were held constant at their means, the median duration for mallard morning recesses was 133.08 [124.80, 141.75] minutes and for mallard afternoon recesses was 134.90 [127.32, 143.28] minutes (Table 1). The median duration for gadwall morning recesses was 111.75 [104.61, 119.34] minutes and for gadwall afternoon recesses was 170.64 [161.17, 180.21] minutes (Table 1). Recess duration decreased as the season progressed (linear day of year *β* = 1.00 ± 0.0014, *F*_1,1777.9_ = 9.99, *p* < .01, Figure 4a). Recess duration increased 3% with every 1°C increase in ambient temperature (ambient temperature *β* = 1.03 ± 0.0021, *F*_1,14526.82_ = 227.99, *p* < .0001, Figure 4b). The influence of recess start time on recess duration differed among species (sine‐min × species_MALLARD_ *β* = 0.82 ± 0.02, *F*_1,16109.82_ = 136.36, *p* < .0001, cosine‐min × species_MALLARD_ *β* = 0.89 ± 0.36, *F*_1,15697.59_ = 25.83, *p* < .0001, Figure 4e). Gadwall recesses were shortest between 00:00 and 07:00, steadily increased between 07:00 and 16:00, and then decreased in the late afternoon and evening. Mallard recess duration exhibited an almost inverse pattern and was longest between 00:00 and 07:00, decreased between 07:00 and 12:00, and steadily increased through the afternoon and evening (Figure 4e). Recess duration did not differ among species (*F*_1,2291.33_ = 0.11, *p* = .74) and did not vary with incubation day (*F*_1,4123.30_ = 0.83, *p* = .36, Figure 4c) or with clutch size (*F*_1,1619.26_ = 2.82, *p* = .09, Figure 4d).

### 3.5 Repeatability of recess timing

#### 3.5.1 Within‐nest consistency of recess timing

For each nest with multiple days of morning or afternoon recesses recorded (*N* = 17,163 recesses at 912 nests), 53% of recesses (*N* = 9,081 recesses) began within 60 min of the mean morning or mean afternoon recess start time for that nest (31%; *N* = 5,237 began within 30 min). Hens initiated afternoon recesses more consistently than morning recesses. Forty‐six percent (*N* = 2,999 of 6,486 morning recesses) of morning recesses began within 60 min of the mean morning recess start time for that nest (27%; *N* = 1,733 began within 30 min). Fifty‐seven percent (*N* = 6,040 of 10,677 afternoon recesses) of afternoon recesses began within 60 min of the mean afternoon recess start time for that nest (32%; *N* = 3,462 began within 30 min).

Fifty‐two percent (*N* = 8,958 of 17,163) of recesses ended within 60 min of the mean morning or mean afternoon recess end time for that nest (32%; *N* = 5,495 ended within 30 min). As with start times, afternoon recess end times were more consistent than were morning recess end times. Forty‐seven percent (*N* = 3,062 of 6,486 morning recesses) of morning recesses ended within 60 min of the mean morning recess end time for that nest (28%; *N* = 1,814 ended within 30 min). Fifty‐five percent (*N* = 5,874 of 10,677 afternoon recesses) ended within 60 min of the mean afternoon recess end time for that nest (34%; *N* = 3,661 ended within 30 min).

Sixty‐nine percent (*N* = 11,862 of 17,163) of recess durations were within 60 min of the mean recess duration for that nest (43%; *N* = 7,413 were within 30 min). The durations of morning recesses were more consistent than the durations of afternoon recesses. Seventy‐three percent (*N* = 4,745 of 6,486) of morning recess durations were within 60 min of the mean morning recess duration for that nest (48%; *N* = 3,114 were within 30 min), whereas only 66% (*N* = 7,081 of 10,677) of afternoon recess durations were within 60 min of the mean afternoon recess duration for that nest (40%; *N* = 4,269 were within 30 min).

**Figure 5:**
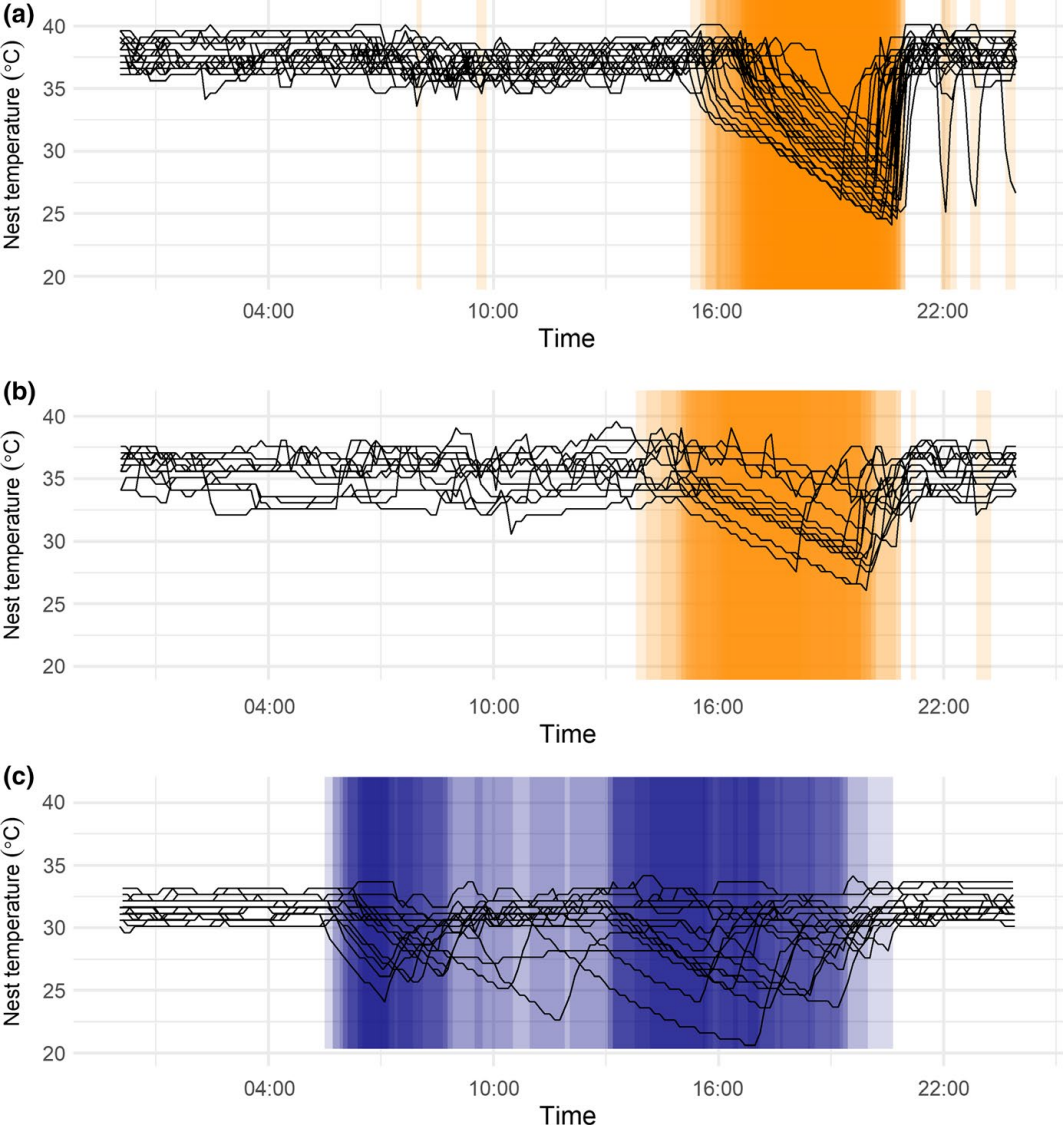
Nest temperature at mallard (blue) and gadwall (orange) nests at the Grizzly Island Wildlife Area, Suisun Marsh, California, 2015–2017 over multiple nest‐days. Each black line represents one nest‐day. Shaded areas indicate recesses, and darker shading represents recesses occurring at the same time of day over multiple days (darker shading = more recesses). Panels show a) one gadwall nest over 31 nest‐days showing highly repeatable afternoon recess timing, b) one gadwall nest over 15 nest‐days showing less‐repeatable afternoon recess timing, and c) one mallard nest over 18 nest‐days showing not‐repeatable recess timing

Figure 5 illustrates consistency of recess timing across multiple nest‐days for three nests.

#### 3.5.2 Intraclass correlation scores for repeatability of recess timing and duration

Repeatability estimates and the corresponding estimates of fixed effects variance are reported in Table 3. Adjusted repeatability estimates (ICC) ranged from 0.06 to 0.35 for recess start time. Recess start time was more repeatable for morning recesses than for afternoon recesses, for mallard and gadwall both together (ICC = 0.29 ± 0.02 for morning recesses vs. 0.23 ± 0.01 for afternoon recesses) and when examined separately (ICC = 0.30 ± 0.02 mallard morning recess vs. 0.26 ± 0.02 mallard afternoon recess; 0.25 ± 0.03 gadwall morning recess vs. 0.18 ± 0.02 gadwall afternoon recess). Repeatability estimates for recess start time were highest (i.e., individual differences were most consistent) for nest‐days on which only one recess took place (ICC = 0.32–0.35). Similarly, recess end times were more repeatable for morning recesses than for afternoon recesses (for morning recesses, both species together ICC = 0.32 ± 0.02, mallard ICC = 0.32 ± 0.02, gadwall 0.30 ± 0.03; for afternoon recesses, both species together ICC = 0.17 ± 0.01, mallard ICC = 0.20 ± 0.02, gadwall 0.12 ± 0.01), and repeatability estimates for recess end time were highest for nest‐days with only one recess (both species together ICC = 0.41 ± 0.02, mallard ICC = 0.43 ± 0.02, gadwall 0.34 ± 0.02). Repeatability estimates were lower for recess duration than for recess start and end time (ICC = 0.09–0.29). Repeatability estimates for recess duration were highest for nest‐days with only one recess (ICC = 0.16–0.29).

**Table 3:** Estimated intraclass correlation coefficients (ICC) ± standard error (*SE*) for recess start time, recess end time, and recess duration within versus among nests, for mallard and gadwall nesting in Suisun Marsh, Grizzly Island Wildlife Area, California, 2015–2017

Model Fixed effects^a^	Species^b^	Nest‐days^c^	Recesses^d^	Enhanced agreement repeatability ICC (*SE*)^e^	Var_FE_ (*SE*)^f^	Adjusted repeatability ICC (*SE*)^g^
Recess start time						
Species + Morning/Afternoon	Both	All	All	0.01 (0.00)	0.82 (0.00)	0.06 (0.01)
Species	Both	All	Morning	0.29 (0.02)	0.00 (0.00)	0.29 (0.02)
Species	Both	All	Afternoon	0.23 (0.01)	0.00 (0.00)	0.23 (0.01)
Species	Both	Single recess	All	0.32 (0.02)	0.08 (0.01)	0.35 (0.02)
Morning/Afternoon	Mallard	All	All	0.01 (0.00)	0.82 (0.00)	0.06 (0.01)
	Mallard	All	Morning			0.30 (0.02)
	Mallard	All	Afternoon			0.26 (0.02)
	Mallard	Single recess	All			0.33 (0.02)
Morning/Afternoon	Gadwall	All	All	0.01 (0.00)	0.80 (0.00)	0.06 (0.01)
	Gadwall	All	Morning			0.25 (0.03)
	Gadwall	All	Afternoon			0.18 (0.02)
	Gadwall	Single recess	All			0.32 (0.02)
Recess end time						
Species + Morning/Afternoon	Both	All	All	0.04 (0.00)	0.65 (0.00)	0.12 (0.01)
Species	Both	All	Morning	0.32 (0.02)	0.01 (0.00)	0.32 (0.02)
Species	Both	All	Afternoon	0.16 (0.01)	0.04 (0.01)	0.17 (0.01)
Species	Both	Single recess	All	0.36 (0.02)	0.13 (0.01)	0.41 (0.02)
Morning/Afternoon	Mallard	All	All	0.05 (0.00)	0.65 (0.00)	0.14 (0.01)
	Mallard	All	Morning			0.32 (0.02)
	Mallard	All	Afternoon			0.20 (0.02)
	Mallard	Single recess	All			0.43 (0.02)
Morning/Afternoon	Gadwall	All	All	0.03 (0.00)	0.63 (0.01)	0.09 (0.01)
	Gadwall	All	Morning			0.30 (0.03)
	Gadwall	All	Afternoon			0.12 (0.01)
	Gadwall	Single recess	All			0.34 (0.02)
Recess duration						
Species + Morning/Afternoon	Both	All	All	0.10 (0.01)	0.11 (0.00)	0.11 (0.01)
Species	Both	All	Morning	0.20 (0.01)	0.03 (0.01)	0.20 (0.01)
Species	Both	All	Afternoon	0.16 (0.01)	0.02 (0.00)	0.16 (0.01)
Species	Both	Single recess	All	0.24 (0.01)	0.02 (0.01)	0.24 (0.01)
Morning/Afternoon	Mallard	All	All	0.14 (0.01)	0.06 (0.00)	0.15 (0.01)
	Mallard	All	Morning			0.20 (0.02)
	Mallard	All	Afternoon			0.21 (0.01)
	Mallard	Single recess	All			0.29 (0.02)
Morning/Afternoon	Gadwall	All	All	0.05 (0.01)	0.18 (0.01)	0.07 (0.01)
	Gadwall	All	Morning			0.18 (0.02)
	Gadwall	All	Afternoon			0.09 (0.01)
	Gadwall	Single recess	All			0.16 (0.02)

a Fixed effects accounted for in repeatability estimation. Day of year (linear and quadratic terms) is included in all models.

b Species included in data.

c Nest‐days included in data. “Single recess” indicates nest‐days on which only one recess occurred.

d Recesses included in data. “Morning” indicates only recesses that started prior to 12:00 for each nest‐day, etc.

e Repeatability with fixed effect variance included in the denominator of the estimate. All associated *p* < .0001 (*α* = 0.05). Enhanced agreement repeatability = adjusted repeatability when no fixed effects are included in the model.

f Proportion of the total variance attributable to fixed effects.

g Repeatability after controlling for fixed effects (i.e., fixed effects held to zero). All associated *p* < .0001 (*α* = 0.05).

Fixed effects (species and morning/afternoon) accounted for most of the variance in recess start and end times, and when only day of year was included in the model (i.e., estimates did not control for variation attributable to other fixed effects), ICC estimates increased (Table 3). Fixed effect variance still accounted for some variation in recess timing and duration, yet the contribution was much smaller for recess duration than for recess start and end times. All repeatability values were significantly different from 0 (likelihood‐ratio tests, all *p* < .0001).

#### 3.5.3 Probability of incubation recess

**Figure 6:**
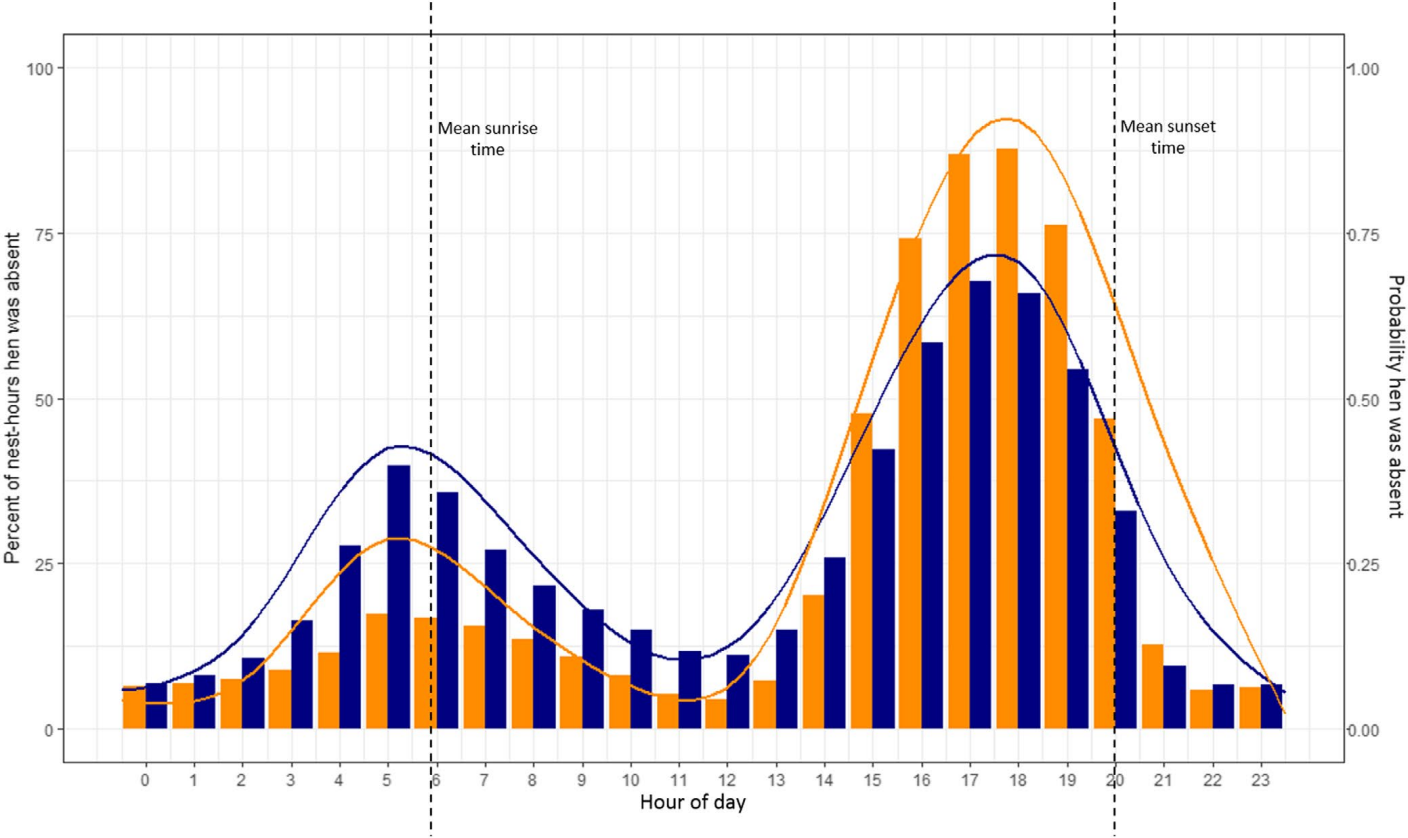
Timing of hen absence from the nest by hour for gadwall (orange bars, orange line) and mallards (blue bars, blue line) nesting at the Grizzly Island Wildlife Area, Suisun Marsh, California, 2015–2017. Bars represent the observed percent of nest‐hours during which a hen is absent from the nest by species. Lines represent the predicted probability that a hen is absent from the nest based on a binomial generalized linear mixed model (GLMM) which included circular hour of the day (using sine‐hr, cosine‐hr, and their 2x frequency variables), species, day of year (linear and quadratic terms), and incubation day as fixed effects, and nest identification and year as random effects. Interactions between species and all other variables were also included. “Mean sunrise time” and “Mean sunset time” represent the mean local sunrise and sunset times

The highest proportions of recesses occurred between 16:00 and 19:00, whereas hens were present on the nest most consistently between 22:00 and 01:00 (Figure 6). The timing of nest absence differed among species and was influenced by the time of day (sine‐hr × species_MALL_ odds = 0.40, *χ* ^2^ = 4,151.90, *p* < .0001; cosine‐hr × species_MALL_ odds = 1.10, *χ* ^2^ = 26.74, *p* < .0001; sine‐hr2x × species_MALL_ odds = 0.97, *χ* ^2^ = 3.05, *p* = .08; cosine‐hr2x × species_MALL_ odds = 0.67, *χ* ^2^ = 507.68, *p < *.0001, Table 4). For example, gadwall hens were 2.5 times more likely to be present at the nest (rather than absent) at 05:00, while mallard hens were 1.4 times more likely to be present at the nest (Figure 6) at 05:00. Gadwall hens were 7.3 times more likely to be absent from the nest at 17:00 than present, while mallard hens were 2.4 times more likely to be absent from the nest at 17:00 than present (Figure 6). The odds of hens being present on the nest at any given hour of the day increased with incubation day (odds = 1.01, *χ* ^2^ = 13.31, *p < *.0005), but did not vary with day of year (odds = −1.00, *χ* ^2^ = 3.28, *p* = .07; day of year^2^ odds = 1.00, *χ* ^2^ = 0.35, *p* = .55).

**Table 4:** Summary of results from generalized linear mixed model (GLMM) predicting the probability of absence from the nest by hour for mallard and gadwall nesting at Suisun Marsh, Grizzly Island Wildlife Area, California, 2015–2017

	Estimate (Odds)	*SE*	*χ* ^2^ (1)	*p*‐value
Incubation day^a^	1.01	0.0022	13.31	<.001
Species_MALLARD_	0.68	0.0326	64.16	<.0001
Day of year	1.00	0.0018	3.28	.07
Day of year^2^	1.00	0.0000	0.35	.55
Sine‐hr	4.17	0.0474	15,801.53	<.0001
Cosine‐hr	1.43	0.0227	13,512.68	<.0001
Sine‐hr2x	0.69	0.0088	834.28	<.0001
Cosine‐hr2x	5.36	0.0775	13,512.68	<.0001
Incubation day × Species_MALLARD_	1.00	0.0026	3.63	.06
Day of year × Species_MALLARD_	1.00	0.0021	0.61	.44
Day of year^2^ × Species_MALLARD_	1.00	0.0000	3.71	.05
Sine‐hr × Species_MALLARD_	0.40	0.0057	4,151.90	<.0001
Cosine‐hr × Species_MALLARD_	1.11	0.0215	26.74	<.0001
Sine‐hr2x × Species_MALLARD_	0.97	0.0154	3.05	.08
Cosine‐hr2x × Species_MALLARD_	0.67	0.0192	507.68	<.0001

Note

Model includes nest identification and year as random effects.

a Days after clutch completion.

## 4 DISCUSSION

Nest attendance plays an important role in determining nest survival (Aldrich & Raveling 1983; Prop et al., 1984), and the timing of nest attendance and recesses reflects the need of incubating parents to both maintain proper physical conditions for embryonic development and meet their own metabolic needs (Reid et al., 2002; Tinbergen & Williams, 2002). Our results demonstrate the influence of various ecological parameters on the timing of incubation recesses in mallard and gadwall and illustrate key differences in nesting ecology between species. The frequency, timing, and/or duration of incubation recesses changed with the day of year, incubation day, and ambient temperature at the nest, and these relationships varied between mallard and gadwall. Furthermore, we found that the timing and duration of recesses were repeatable within individual hens, and that most absences from the nest occurred either shortly after dawn, or in the late afternoon, ending before sunset.

The number of recesses that gadwall hens in Suisun Marsh took per day increased as mean daily ambient temperature increased, whereas the number of recesses that mallard hens took per day decreased as mean daily ambient temperature increased. This effect, however, was small for both species and was unlikely to have a biologically important effect. Similarly, the number of recesses that gadwall took per day increased as incubation progressed, and the number of recesses that mallard took per day decreased, but this effect was very small for both species even over the total incubation period and was unlikely to have a biologically important effect. Mallard were more likely to take two recesses each day, one recess each in the morning and afternoon, than were gadwall, which were more likely to take a single recess in the afternoon. Our results are similar to those reported for mallard nesting in the Prairie Pothole Region, where recess rates were 1.72 recesses per day (range 0–4, Hoover, 2002), but lower than observed in a study of aviary‐confined mallard in Manitoba (3.3 recesses per day; Caldwell & Cornwell, 1975). Conversely, our results differed from those reported for gadwall nesting in the Prairie Pothole Region, which took 2.2 recesses per day on average (Lorenz, 2005). Gadwall in the Prairie Pothole Region also substantially increased their recess frequency as incubation progressed, but took fewer recesses under warmer ambient temperature conditions (Lorenz, 2005).

In Suisun Marsh, recesses started earlier in the day on days that ambient temperatures were warmer, and ambient temperature affected recess start time more for gadwall than for mallard (Figure 2). Hens may recess earlier on days that ambient temperatures are warmer and later on days that ambient conditions are cooler due to their needs to both forage and to limit the rate of egg cooling while they are away from the nest, which may be greater under cooler ambient conditions. Because both the eggs and the hens themselves are subject to ambient temperatures, the relationship between recess timing and ambient temperature may also be shaped by hens acting to meet their own thermoregulatory needs (e.g., cooling themselves by wetting their feathers during recesses when ambient temperatures are warmer) in addition to those of the eggs.

Recesses ended earlier as ambient temperature increased (when controlling for time of day), and the relationship between recess start time and recess end time differed among species such that mallard both started and ended their earliest and latest recesses earlier than did gadwall (Figure 4e). Recesses occurring around dawn or dusk, when light conditions were low, ended earlier with later start times, but recesses that started during full daylight ended later with later start times. Recesses also ended later as the breeding season progressed. Together, this suggests that seasonal differences in recess end time were driven by seasonal differences in day length. To our knowledge, only one other study has reported the timing of dabbling ducks’ return to the nest following a recess (morning recesses only, Gloutney et al., 1993). Dabbling ducks nesting in central Saskatchewan ended morning recesses at times similar to hens in this study (mallard 06:34 ± 02:27, gadwall 07:53 ± 02:45, Gloutney et al., 1993; mallard 08:24 ± 03:37, gadwall 08:33 ± 03:03 this study; we note that one hour difference is attributable to differential application of daylight savings time).

Impending darkness associated with sunset appeared to have a large effect on mallard and gadwall incubation behavior. Hens taking late afternoon recesses returned to their nests earlier than expected when the sunset occurred prior to their expected return time, and many recesses that would otherwise have been expected to end after sunset ended prior to sunset. This indicates that hens avoided returning to the nest when ambient light conditions were low. At least two factors may contribute to this pattern: (a) nests may be difficult to find in low‐light conditions, because birds often use visual spatial cues to return to specific locations (e.g., food resources; Croston et al., 2016) and nest relocation for waterfowl likely relies on visual spatial cues, and/or (b) hens may avoid excess activity near their nests at night, when risk of mammalian nest depredation is highest (Croston, Ackerman, et al., 2018). In Suisun Marsh, a large proportion of duck nest depredation by mammals occurs shortly after sunset (Croston, Ackerman, et al., 2018). By avoiding activity at or around the nest at this time, hens may reduce the likelihood that their nest is found by a predator (e.g., Martin, Scott, & Menge, 2000).

Recess duration varied with time of day and in both species increased with date and ambient temperature. After accounting for ecological parameters, morning recesses were shorter for gadwall than for mallard (gadwall 111.75 [104.61, 119.34] minutes vs. mallard 133.08 [124.80, 141.75] minutes), whereas afternoon recesses were longer for gadwall than for mallard (gadwall 170.64 [161.17, 180.21] minutes vs. mallard 134.90 [127.32, 143.28] minutes). Recess durations for mallard and gadwall in Suisun Marsh were similar to those of mallard (138.78 ± 104.81 min, Hoover, 2002) and gadwall (179.8 ± 133.80 min, Lorenz, 2005) nesting in North Dakota, but substantially different from those observed for aviary‐housed mallard nesting in southern Manitoba (24 ± 17.5 min, Caldwell & Cornwell, 1975). Recess duration increased with ambient temperature in both our study and in the southern Manitoba aviary‐housed population (Caldwell & Cornwell, 1975). Ambient temperature plays an important role in determining both recess duration and frequency for black ducks (*Anas rubripes,* Ringelman et al., 1982), but it does not appear to drive recess timing for ring‐necked ducks (*Aythya collaris*, Hohman, 1986). Duck eggs are less robust to high temperature than to low temperatures (Webb, 1987); nonetheless, mallard nesting at Suisun Marsh took longer incubation recesses at higher ambient temperatures.

We found that an individual hen's recess timing was consistent across days. Nearly half of morning and afternoon recesses were observed to occur within 60 min of that individual hen's mean. The timing of morning recesses was more repeatable than the timing of afternoon recesses, and mallard recess behavior was more repeatable than gadwall recess behavior (Table 3). Furthermore, a hen's recess behavior was more repeatable on days in which they took only one recess compared to on days in which they took multiple recesses (Table 3). Because repeatability is computed as a ratio of among‐individual variation to total variation within a population, the consistency of an individual hen's recess timing cannot be inferred from repeatability scores alone. By pairing measures of behavioral consistency with repeatability, however, we can attribute repeatability scores to relatively high intraindividual consistency rather than population‐level dissimilarity in recess timing. If hens favor consistent recess timing and prefer to take recesses at one or two “best” times of day, our results suggest that gadwall are more likely to alter recess timing in responses to environmental conditions, while mallard are more robust to variable nesting conditions. The more repeatable timing of morning recesses compared to afternoon recesses may indicate relatively more stressful conditions while incubating overnight (e.g., longer incubation bout, cooler ambient temperatures, and increased exposure to predators) versus during daylight.

Our results identify important factors influencing the timing of incubation recesses in dabbling ducks and provide insight into the ways in which hens balance their needs with those of their developing embryos. The timing of incubation recesses also has important implications for investigators and habitat managers seeking to minimize the impact of observers on nest fate (Livezey, 1980), while also minimizing the likelihood of failing to find active nests (Gloutney et al., 1993). We found that hens were most likely to be away from nests between 04:00 and 07:00 and between 16:00 and 19:00. Therefore, investigators who are interested in flushing a female from the nest should begin searches after 07:00 and should end before 16:00. Because hens typically return to their nests before sunset, flushing hens earlier in the afternoon may afford them more time to complete recess activities and return to the nest, thereby minimizing the impact of nest visits or habitat management activities on nest attendance.

## CONFLICT OF INTEREST

None declared.

## AUTHOR CONTRIBUTIONS

C. L. F., J. T. A., and M. L. C. contributed substantial resources and initiated the project. R. C., C. A. H., M. P. H., and J. T. A. conceived of the idea and designed the study. R. C., C. A. H., M. P. H., and J. T. A. developed and designed the methods. J. T. A., M. P. H., C. A. H., and M. L. C. collected the data. R. C., C. A. H., M. P. H., M. L. C., and J. T. A. curated the data. R. C., C. A. H., M. P. H., and J. T. A. analyzed the data and wrote the paper. All authors edited the paper.

## ETHICAL APPROVAL

Research was conducted with the approval of the U.S. Geological Survey Western Ecological Research Center's Animal Care and Use Committee.

## Data Availability

The raw data in this manuscript are available in ScienceBase (https://doi.org/10.5066/P981DMHZ).
